# Collaborative relation annotation and quality analysis in Markyt environment

**DOI:** 10.1093/database/bax090

**Published:** 2017-12-05

**Authors:** Martín Pérez-Pérez, Gael Pérez-Rodríguez, Florentino Fdez-Riverola, Anália Lourenço

**Affiliations:** ESEI–Department of Computer Science, University of Vigo, Edificio Politécnico, Campus Universitario As Lagoas S/N 32004, Ourense, Spain; CINBIO–Centro de Investigaciones Biomédicas, University of Vigo, Campus Universitario Lagoas-Marcosende, 36310 Vigo, Spain; CEB–Centre of Biological Engineering, University of Minho, Campus de Gualtar, 4710-057 Braga, Portugal

## Abstract

Text mining is showing potential to help in biomedical knowledge integration and discovery at various levels. However, results depend largely on the specifics of the knowledge problem and, in particular, on the ability to produce high-quality benchmarking corpora that may support the training and evaluation of automatic prediction systems. Annotation tools enabling the flexible and customizable production of such corpora are thus pivotal. The open-source Markyt annotation environment brings together the latest web technologies to offer a wide range of annotation capabilities in a domain-agnostic way. It enables the management of multi-user and multi-round annotation projects, including inter-annotator agreement and consensus assessments. Also, Markyt supports the description of entity and relation annotation guidelines on a project basis, being flexible to partial word tagging and the occurrence of annotation overlaps. This paper describes the current release of Markyt, namely new annotation perspectives, which enable the annotation of relations among entities, and enhanced analysis capabilities. Several demos, inspired by public biomedical corpora, are presented as means to better illustrate such functionalities. Markyt aims to bring together annotation capabilities of broad interest to those producing annotated corpora. Markyt demonstration projects describe 20 different annotation tasks of varied document sources (e.g. abstracts, twitters or drug labels) and languages (e.g. English, Spanish or Chinese). Continuous development is based on feedback from practical applications as well as community reports on short- and medium-term mining challenges. Markyt is freely available for non-commercial use at http://markyt.org.

**Database URL:** http://markyt.org

## Introduction

The availability of large, manually annotated text corpora is highly desirable for the development of text mining methods and the robust evaluation and comparison of alternative approaches. Typically, the production of semantically annotated corpora is resource and time consuming, requiring the preparation of robust domain/problem-specific annotation guidelines and interaction with multiple experts. The corpus production workflow may be adapted to the specificities of a given domain of application, but there are common issues to attend to, such as transduction into and out of different formats as well as execution of multiple annotation rounds of multiple annotators with evaluations for consistency at several points. The use of annotation standards and well-established formats are determinant in ensuring corpus sharing and interchangeability. In turn, the key to ensure the quality of the corpus is to actively monitor inconsistencies throughout the rounds of annotation and resolve the most critical issues as soon and as effectively as possible.

Markyt (originally named Marky) is a web-based document annotation tool equipped to manage the production of high-quality annotated corpora [1]. Underlying design principles include (i) general purpose application, i.e. domain specifications are considered only in project configuration and do not affect the general behaviour of the software, (ii) modular and flexible architecture, which enables seamless component extension, (iii) user-friendly and continuously improved interface for human curators, and (iv) powerful analytical abilities that enable corpus quality assessment throughout the whole production cycle. As key features, Markyt enables the creation of multi-user and multi-round annotation projects and implements analytical functionalities for assessing the consistency of the annotations of individual annotators throughout time and inter-annotator agreement (IAA) comprehensively. Regardless of the specifics of each project, the main objectives are to reach a harmonized interpretation of the annotation guidelines among human curators and to be able to achieve an annotator consensus, i.e. produce a final, high-quality version of the corpus.

In the first release of the software, annotation functionalities centred on tagging of entity or concept mentions, enabling the flexible definition of entity classes/types, making the annotation as ergonomic as possible for human curators and supporting annotation quality analysis at the entity level. Since then, Markyt has been used in several practical applications. For example, in the construction of antimicrobial peptide–drug combination networks [2], the exploration of *Pseudomonas aeruginosa* quorum sensing inhibitors [3], and the visualization, prediction and benchmarking of chemical and gene entity annotations at BioCreative V CHEMDNER challenge [4]. Overall, these experiences enabled the identification of desirable refinements and priority extensions.

The logical next step in terms of software extension was to develop annotation and analysis capabilities so that the tool could accommodate the flexible and customized description of relations among entities. Beyond visual appearance/impact, this extension implied the design of annotation perspectives that with a minimal number of clicks could link multiple entities in various, different ways and the refactoring of Markyt’s data model so that it is capable of managing comprehensive statistics about the entities and the relationships produced by each annotator at each round of annotation. Moreover, previous user feedback suggested some improvements to the annotation interface, regarding annotation search, filtering and global/individual editing.

Parallel to this exercise of learning from experience and feedback, the inspection of other annotation tools also provides interesting suggestions for further development. Granted that annotation tools may differ in different technical and functional aspects, the aim of this comparison is primarily 2-fold: the supported data formats, the ergonomics of the visual annotation environment and the scope of analytical capabilities. A detailed comparative table is presented in Supplementary Material S1.

The aim of this paper is thus to describe the new release of Markyt open source platform, in particular, the new annotation perspectives and the enhanced analysis capabilities. To this end, the following sections provide technical details about software development and showcase the functionalities through demos inspired by public biomedical corpora.

## Materials and methods

**Figure 1.**
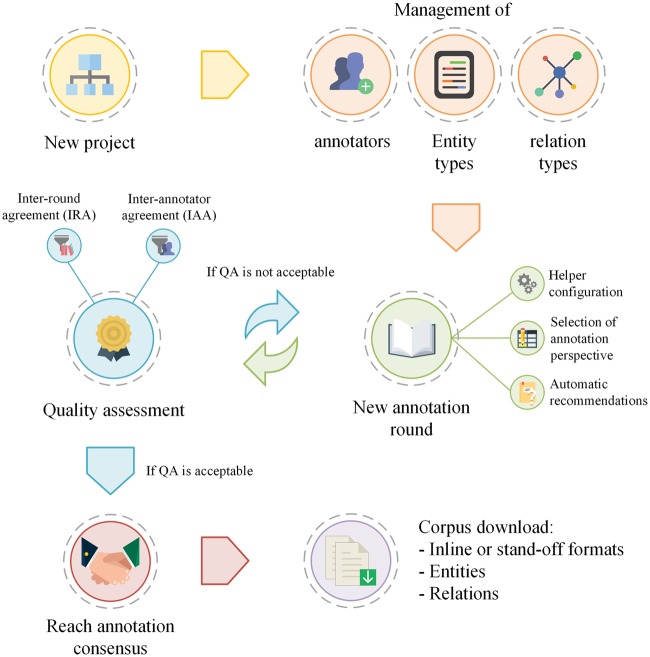
General life cycle of an annotation project in Markyt. At first, the administrator has to describe the project of annotation (i.e. users and annotation types). Then, the process is iterative, i.e. rounds of annotation will be created to enable the multi-user annotation of documents and the quality of the sets of annotations produced will be compared, issuing a new round of annotation till quality is considered acceptable.

Markyt annotation environment tries to accommodate the particular requirements of each project as best as possible while maintaining a general production life cycle (Figure 1). As with similar environments, Markyt requires the specification of the documents to be annotated and the types of entities and relations to be considered (as stated in the project’s guidelines) and enables the participation of one or more annotators in the project. Annotation rounds reflect the iterative nature of the production life cycle. The initial round may consider the raw, unannotated documents or a first set of annotations, based on dictionary lookup or the predictions of external automatic systems. The creation of new rounds of annotation will depend on the evaluation of the quality of existing annotations, namely IAA. Markyt allows the possibility to copy and duplicate rounds. The operation of copy annotations enables a second annotator to work over the annotations of the annotator in an independent way, i.e. the second annotator is able to amend the annotations proposed by the first annotator as desired. In turn, the operation of duplicate annotations is a means to create a data checkpoint, i.e. to allow rollback if needed. For example, it may be handy when the annotation guidelines suffer a significant change, but the new round of annotation shows that such change was detrimental to the overall annotation purposes. Once corpus quality is considered acceptable (administrative decision), the final corpus is produced based on user-specified annotation consensus rules (administrative decision) and becomes available for download.

Markyt is built on top of open technologies and standards to grant extensibility and interoperability with other systems. Its web model-view-controller (MVC) design pattern and core development are supported by the open-source CakePHP framework [5]. User interface relies on HTML5 (http://www.w3.org/TR/html5/) and CSS3 technologies (http://www.css3.info/). Rangy library assists in browser-independent implementation of common document object model (DOM) range and selection tasks (http://code.google.com/p/rangy/), and Ajax and JQuery technologies are used to enhance user–system interaction (http://jquery.com/).

The following subsections describe the rationale behind the new functionalities and the basics of their implementation.

### Relation identification and extraction

The majority of biomedical analyses that resort to the mining of scientific (or other sources of) literature ultimately aim to acquire some sort of categorized/typified and structured representation of the most meaningful mentions to domain relations. Thus, text mining challenges are pushing forward increasingly ambitious recognition tasks, both in terms of the complexity of the information to be extracted and the biomedical semantics scopes covered [6–10].

The extraction of domain relations implies the identification of the involved entities and the description of the relations among them. The granularity of the relations is important for annotation purposes. It may be interesting to annotate relations at a document level, e.g. co-occurring entities in abstracts or full-texts, or at the mention level, i.e. relations between specific entity mentions within particular contexts. Moreover, various types of relations may be considered (e.g. different domain semantics, including directionality), and entities may act as direct participants (e.g. regulated/regulator genes, drug–gene interactions or reaction substrates/products) or supporting participants (e.g. cause/symptom, location/site or experimental condition).

**Figure 2.**
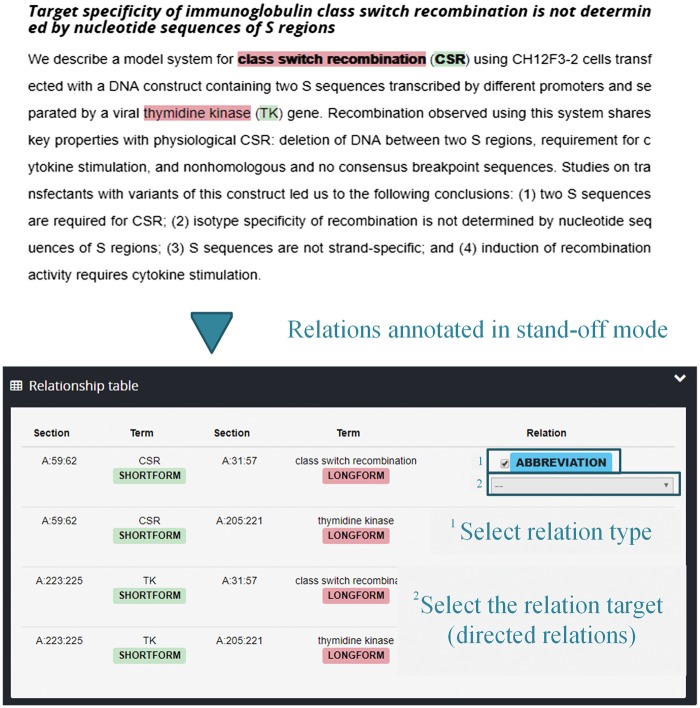
Illustration of the Markyt tabular annotation perspective for entities and relations.

Markyt annotation environment enables the inline annotation of entity mentions and provides two annotation perspectives, one inline with the text and another in a stand-off, tabular mode. Figure 2 shows an example of the stand-off annotation perspective. For more information about these perspectives (including how the entities and relations are entered in the system), see Supplementary Material S2.

It is important to remark that the perspectives of annotation are interchangeable at the project level if and only if the whole annotated relations are deleted, i.e. relations annotated at the document level need to be properly located in the text.

### Quality assessment

**Figure 3.**
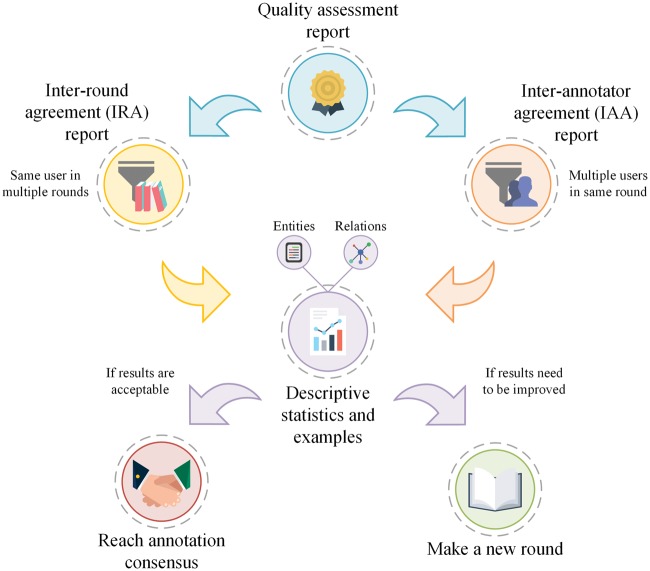
Production life cycle of the quality assessment of intra-annotator consistency throughout time (IRA) and IAA. Markyt allows the execution of different iterations of rounds in order to achieve the desired quality.

Markyt quality assessment module is responsible for analysing the consistency of the inter-round agreement (IRA) for a single annotator as well as the IAA for each round of annotation of multiple annotators (Figure 3). Individual consistency checking is important to know whether the annotator has significantly changed the annotation pattern throughout time, and typically after guidelines revision or round debates. IAA provides critical information about the overall quality of the corpus, evidencing annotation types that are more subjective, i.e. prone to discrepancies among experts.

The rates of agreement among annotators or among rounds are calculated. In particular, the standard measures of recall, precision and *F*-score are calculated as follows [15–17]:

$$F-score=2\times \frac{\text{precision}\times \text{recall}}{\text{precision}+\text{recall}}$$

$$\text{precision}=\frac{\text{number}\;\text{of}\;\text{identical}\;\text{annotations}\;\text{in}\;\text{set}\;A\;\text{and}\;\text{set}\;B}{\text{number}\;\text{of}\;\text{annotations}\;\text{in}\;\text{set}\;A}$$

$$\text{recall}=\frac{\text{number}\;\text{of}\;\text{identical}\;\text{annotations}\;\text{in}\;\text{set}\;A\;\text{and}\;\text{set}\;B}{\text{number}\;\text{of}\;\text{annotations}\;\text{in}\;\text{set}\;B}$$

Regarding entity annotations, Markyt enables IAA and IRA calculations to be stricter or more relaxed. It is possible to calculate agreement rates based on exact annotation matches, i.e. where text spans identified by a pair of annotators match perfectly, and relaxed annotation matches, i.e. where text spans identified by a pair of annotators overlap with each other by a user-specified, minimum number of characters.

The evaluation of relation annotations follows the same guidelines. Mention-level relation evaluation is based on annotation offsets, i.e. each relation is described by the offsets of the participants (i.e. name and type of the entity, and specific textual references) as well as the relation type. The same relation (i.e. tuple of participants and relation type) may occur several times in the document, but they are differentiated by the specific context of occurrence, i.e. offsets. In turn, document-level relation evaluation analyses relations at the document level, i.e. there are no text offsets, just the participants (name and type of the entity) and the relation type. A given tuple of participants and relation type may only occur one time per document.

At this level, agreement is divided into having established a relation between the same pair of entities and associating or not the same type to the relation.

The iterative annotation process concludes when the rate of IAA is considered acceptable or not possible to improve in further rounds (administrative decision). Then, the administrator(s) establishes the grounds to perform annotation consensus and produce the final corpus. For example, it may be satisfactory to accept all annotations that have been tagged by, at least, half of the annotators or, in other cases, it may only make sense to keep annotations for which all annotators agreed upon.

### Automatic annotation recommendations and additional support

Markyt does not integrate automatic prediction systems, but it does enable the use of annotations from such systems in benefit of the annotation process. In particular, Markyt includes a module capable of automatically producing annotation recommendations based either on prior annotation history (i.e. the mentions annotated previously by the annotator) or derived from existing named entity recognizers (i.e. acting as external annotation providers).

Prior annotation history can be looked at as the ground truth of the annotator. So, by pointing out potential misses (i.e. any text fragment matching one of these annotations and without an annotation), Markyt helps the annotator to debug/check out the consistency of his annotations. On the other hand, by looking into the predictions of automatic recognition systems, the work of the annotator may be facilitated (i.e. just eliminating the unintended annotations).

**Figure 4.**
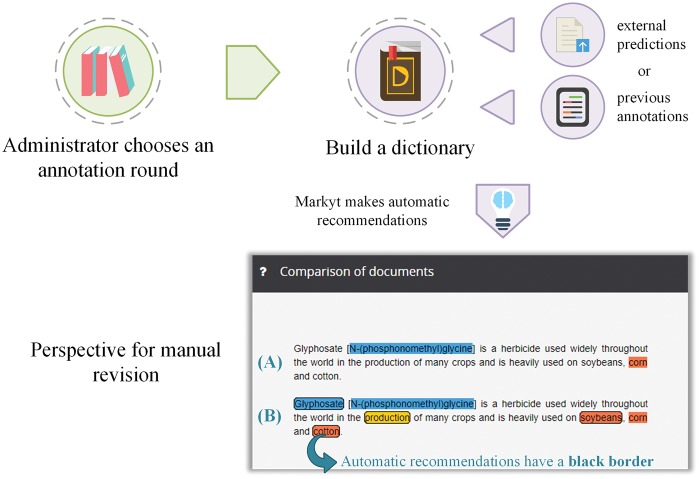
Production life cycle of the automatic annotation recommendations. The administrator can generate recommendations based on annotation history, asset of externally generated annotation predictions or a dictionary. Annotators will manually revise these recommendations (visually differentiated by the use of bordered marks).

Figure 4 illustrates the workflow to generate automatic recommendations and how the annotation environment presents an integrated view of the recommendations, together with the manual annotations, for manual expert revision. Automatic annotations are visually differentiated by the use of bordered marks. Annotators can edit or eliminate recommendations as considered appropriate. To minimize effort, annotators do not have to perform any action to accept an automatic recommendation. At the end of the round, all remaining automatic recommendations will be accepted as valid annotations and therefore will not be differentiated in the next round.

In terms of aiding in manual annotation/revision, Markyt environment has been significantly improved based on practical user feedback. Specifically, new amendment and navigation features have been incorporated in the annotation environment. For example, it is possible to navigate through all the mentions of a given entity in the corpus and to change the type/class of a particular mention or of all similar mentions in the entire corpus. Furthermore, several annotation helpers have been developed to minimize the time and effort of annotation (e.g. automatic trimming of the selected mention or search the term in biomedical databases). For details on the supporting functionalities in the annotation environment, see Supplementary Material S3.

### Extended support of annotation formats

Markyt can import raw, unannotated documents and existing entity and relation annotations. This functionality is useful when taking on existing annotation projects (e.g. extending an entity corpus to a relation corpus) or when the results of automated identification and relation extraction tools are considered useful as the basis of annotation.

Currently, Markyt is able to import raw, unannotated documents in TSV and BioC inline XML [11]. Moreover, the tool is able to import annotations following the formats BRAT standoff annotation [12], BioC inline XML [11], BioNLP standoff representation [13] and BioCreative TSV [14]. Both documents and annotations are stored in the relational database supporting Markyt operations. Document contents are saved in HTML format with UTF-8 encoding which ensures multilingual support. After reaching annotation consensus, users can export the final corpus in Markyt standoff and inline TSV formats, Markyt JSON or BioC XML.

## Results

Demos of various annotation projects are available as part of Markyt documentation (http://www.markyt.org/annotationDemo/). These demos are inspired by public corpora, typically originating in text mining challenges and research projects. The corpora were selected based on their nature and specifics, aiming to show the annotation features and analysis capabilities of Markyt at different levels of complexity. Hence, demos encompass documents of varied source (e.g. PubMed abstracts, PMC full texts, Tweets and clinical notes), different document languages (e.g. English, Spanish or Chinese) and different levels of complexity in entity and relation annotation (e.g. overlapping entities). Moreover, the corpora TAC2017-2U3R was used to artificially recreate the collaborative and iterative corpus production process.

Markyt demos are not editable projects, i.e. any anonymous user can freely inspect them, but no changes are permanently saved. Table 1 describes the available demos. In particular, the project entitled ‘TAC2017-2U3R’ enables the exploration of a multi-user and multi-round setup, where one can look into the analysis capabilities of the software as well as the ability to create annotation rounds and achieve annotation consensus. Specifically, this project is inspired by one of the latest BIONLP corpora, which is devoted to the description of adverse drug reactions in prescription drug labels (https://bionlp.nlm.nih.gov/tac2017adversereactions/). This corpus accounts for six entity annotation types (e.g. ‘factor’, ‘severity’, ‘adverse reaction’ and ‘animal’) and three relation annotation types (i.e. ‘hypothetical’, ‘effect’ and ‘negated’). Our demonstration project considers all the original entity and relation annotation types and emulates the work of two annotators and three rounds of annotation.

**Table 1.** Corpora used for Markyt demonstration purposes

Demo name	Format	Language	Doc type	#Documents	#Types	#Annotations
AB3P [15]	BioC	English	Abstracts	1250	E: 2	E: 2423
					R: 2	R: 1201
TweetADR [16]	TSV	English	Tweets	799	E: 3	E: 560
AIMed [17]	BioC	English	Abstracts	225	E: 1	E: 4236
					R: 1	R: 1000
BCV CDR [6]	BioC	English	Abstracts	500	E: 1	E: 5107
BioADI [18]	BioC	English	Abstracts	1201	E: 2	E: 3402
					R: 1	R: 1691
CellFinder [19]	BioC	English	Full text	10	E: 6	E: 5842
Craft 2.0	BRAT	English	Full-text	67	E: 4	E: 81040
					R: 2	R: 56802
DDI2011 [20]	BioC	English	Abstracts	435	E: 1	E: 11260
					R: 1	R: 2402
Genereg [21]	BioC	English	Abstracts	314	E: 10	E: 6357
					R: 3	R: 1729
GENIA [21]	BioNLP	English	Abstracts	800	E: 11	E: 16185
					R: 10	R: 6447
Grec_ecoli [22]	BioC	English	Abstracts	167	E: 57	E: 6332
					R: 13	R: 3998
Herb-Chemical [23]	BioNLP	English	Abstracts	1.109	E: 3	E: 2815
					R: 2	R: 1194
Mantra [24]	BRAT	Spanish, German, French	European Medicines Agency documents (EMEA)	100, 100, 100	E: 11, 10, 10	E: 349, 348, 351
Medstract [25]	BioC	English	Abstracts	198	E: 2	E: 317
					R: 1	R: 158
MLEE [26]	BioNLP	English	Abstracts	262	E: 45	E: 13810
					R: 15	R: 5396
Phylogeography [27]	BRAT	English	Full-text	28	E: 7	E: 17486
Protein coreference [28]	BioNLP	English	Abstracts	800	E: 2	E: 13427
					R: 1	R: 2143
TAC2017-2U3R [29]	XML	English	Drug labels	101	E: 6	E: 14486
					R: 3	R: 2486
TCMRelationExtraction [30]	TSV	Chinese	Abstracts	20 000	E: 4	E: 5786
TwiMed [31]	BRAT	English	Tweets	695	E: 3	E: 1119
					R: 3	R: 445

E, entities; R, relations.

**Figure 5.**
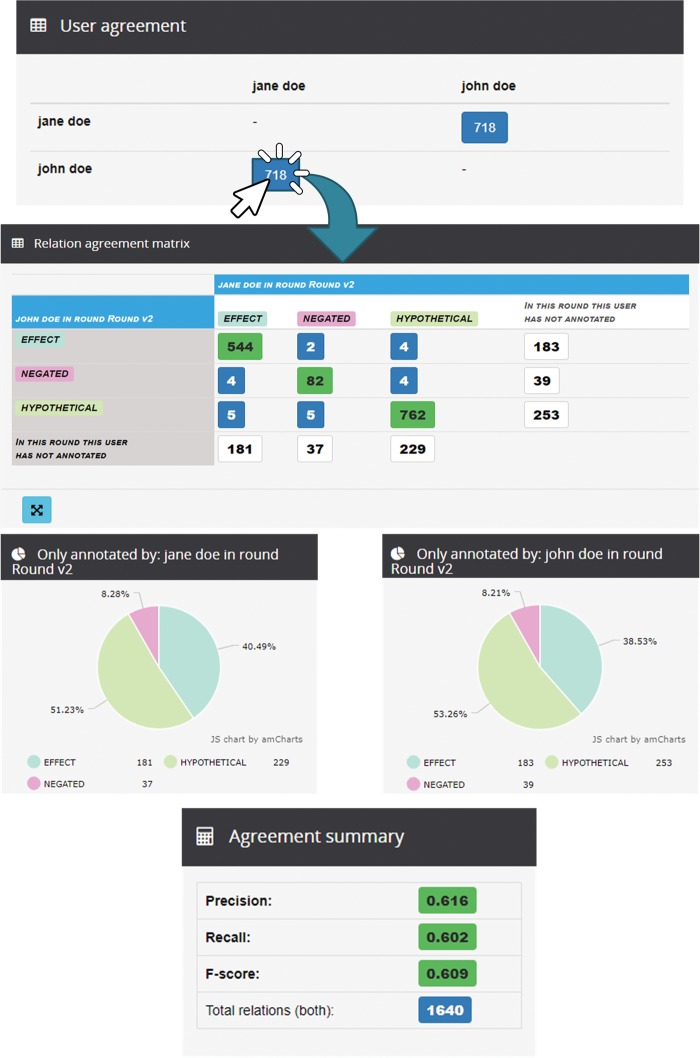
Analysis of IRA for round 2 between Jane and John in the demo project ‘TAC2017-2U3R’.

In this particular project, entity annotations were revised previously and the annotators now focused on the annotation of relations. At the first round, the rate of IAA for relations (*F*-score) is 50%, i.e. 65% of agreement for the type ‘hypothetical’, 67% for ‘effect’ and 51% for ‘negated’. After discussing how to improve these rates, the administrator launched a second round of annotation. Figure 5 illustrates some of the statistics generated at the end of this second round. The IAA for relations (*F*-score in agreement summary) is 61% (an exact value of 0.609), i.e. the rates of IAA were 75%, 74% and 64% for the types ‘hypothetical’, ‘effect’ and ‘negated’, respectively.

These values can be calculated according to the data showed in the relation agreement matrix (true positives, false positives and false negatives) and the *F*-score equation. For example, considering the type hypothetical in round 2, it had 762 true positives, 263 false positives (the sum of the horizontal values) and 237 false negatives (the sum of vertical values). According to this, the type had a precision of 74% and a recall of 76%, so the final *F*-score was the previously commented value of 75%.

**Figure 6.**
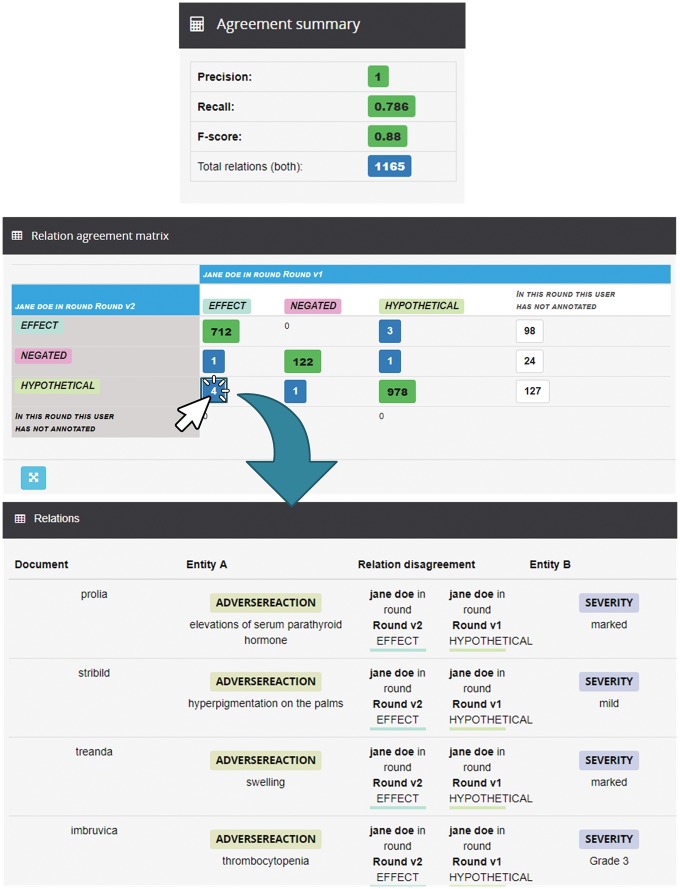
Analysis of IRA for Jane between round 1 and round 2. It shows the Markyt IAA capabilities (e.g. the agreement matrix shows the annotation discrepancies by type or the agreement measures in the summary).

Markyt also enables the analysis of IRA, i.e. the inspection of the work of individual annotators throughout multiple rounds of annotation. Figure 6 shows how Jane progressed in the annotation rounds 1 and 2. In the relation agreement matrix, it is possible to observe the main differences between the two rounds. For example, in round 2, Jane changed the relation type from ‘Effect’ to ‘Hypothetical’ of four different relations. Besides, she annotated 127 new relations of ‘Hypothetical’ type. In the relation annotations table, it is possible to observe in a more detailed way, which were the modified relations. For example, Jane first annotated the relation linking ‘thrombocytopenia’ and ‘Grade 3’ (in document #46) as ‘Hypothetical’ and then changed it to ‘Effect’.

**Figure 7.**
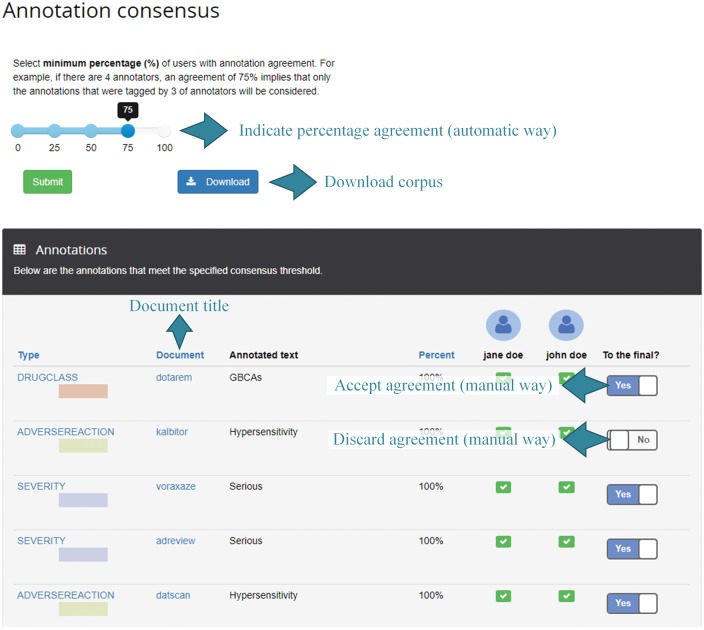
Example of consensus perspective. The percentage of users that have the same annotation is used to decide on the annotations to be included in the consensus corpus. This threshold is configurable as well as to select and discard annotations in a manual way.

The third round of annotation represents the final round for this project. At this point, the administrator proceeds with the generation of annotation consensus and make the corpus available for download. Typically, the annotations should reach a given threshold of agreement among annotators to be part of the final corpus. Figure 7 shows the consensus parameterization and manual revision. For illustration purposes, the annotation ‘Hypersensitivity’ is not considered in the final consensus (the checkbox is set to no) although both annotators annotate it correctly.

The final corpus is available for download in TSV, JSON and BioC formats.

## Discussion

The production of high-quality annotated corpora, essential for knowledge extraction tasks, is challenged by domain-specific semantic ambiguity and inevitable inter-annotator discrepancy. Rarely, annotators agree completely on the set of entities and relations to be annotated and exactly how to annotate them. For this reason, document annotation projects usually involve multiple domain experts, working in an iterative and collaborative manner, so as to guarantee the quality of the final corpus. Often, this process can be time and labour consuming and, therefore, annotation software should provide as much support as possible.

Besides providing a broad-scope and well-equipped annotation environment, Markyt distinguishes itself from similar toolkits in that it enables the comprehensive analysis of intra-annotator and inter-annotator annotation patterns. Markyt helps identify inconsistencies in the work of individual annotators throughout multiple rounds of annotation as well as discrepancies among annotators. In particular, it is able to pinpoint the annotation types and terms (in entities or relations) that raise more conflicts. These insights can be of aid in revising the annotation guidelines (e.g. making the annotators more sensitive to specific semantics) as well as giving specific directions to the annotators.

## Conclusions

Markyt annotation environment is approximately 3 years old and its two initial software development directives persist, i.e. flexible and customizable annotation and comprehensive quality assessment. Development at these two fronts is being prioritized based on community emerging needs as well as feedback coming from the use of Markyt in practical biomedical applications.

This paper describes the latest release of the software, in particular, the implementation of relation annotation at the mention and document levels, the integration of automatic annotation recommendations and the extension of analysis capabilities.

Future directions include exploring parallel computing to enhance the performance of more demanding tasks for the management of larger corpora, managing complex annotation tasks with alternative ways of assigning documents to the team of annotators, extending the capabilities for automatic recommendation and editing modules to relation annotation, and devising new visualization perspectives for rendering large volumes of annotations. 

## Mode of availability

Markyt software, comprehensive documentation of the functionalities and usage demonstrations are freely available for non-commercial use at http://markyt.org

## Authors’ contributions

M.P.-P. and G.P.-R. developed the Markyt software, and A.L. coordinated the project. All authors tested the software and participated in writing of the manuscript.

## Funding

This work was partially supported by the Portuguese Foundation for Science and Technology (FCT) under the scope of the strategic funding of UID/BIO/04469/2013 unit and COMPETE 2020 (POCI-01-0145-FEDER-006684). The authors also acknowledge the PhD grants of M.P.-P. and G.P.-R., funded by the Xunta de Galicia.

## Supplementary data

Supplementary data are available at *Database* Online.

## Supplementary Material

Supplementary Data 1

Supplementary Data 2

Supplementary Data 3
