# Supplementary material for: Collaborative relation annotation and quality analysis in Markyt environment
Source: Database (Oxford). 2017 Dec 5;2017:bax090. doi: 10.1093/database/bax090 (PMC5737204; doi:10.1093/database/bax090)
Supplement: Supplementary Data 1 [file bax090_supplementary_material_1.doc]

*Supplementary material 1*: Comparative table of annotation tools

Collaborative relation annotation and quality analysis in Markyt environment

Martín Pérez-Pérez, Gael Pérez-Rodríguez, Florentino Fdez-Riverola, Anália Lourenço^§^

^§^Corresponding author

| **Tool** | **Initial publication** | **Last update** | **Availability** | **Load annotated documents** | **Input** | **Output** | **^1^Rich text support** | **Customized types and colours** | **Multi-user** | **Search and annotate** | **Annotation comments** | **Relationship annotation** | **Annotation agreement** | **Install** | **Dependencies** |
| --- | --- | --- | --- | --- | --- | --- | --- | --- | --- | --- | --- | --- | --- | --- | --- |
| [A.nnotate](http://a.nnotate.com/) | 2009 | 2017 | Download not available | Y | TXT, WORD, PDF, HTML | PDF, DOCX, XML | Y | N | Y | Y | Y | N | N | N | Web browser |
| [Anotation-Studio](http://www.annotationstudio.org/) | 2013 | 2015 | GNU GPL2 | Y | WORD, PDF, TXT | W3C’s Open Annotation format | Y | N | N | N | Y | N | N | SA, Web | PostgreSQL, Nde.js, NPM, MongoDB |
| [Argo](http://argo.nactem.ac.uk/) | 2012 | 2014 | Download not available | Y | XMI ,BIOC, A1,A2 | BIOC,A1,A2,XMI | N | N | Y | Y | Y | Y (TA) | N | N | Web browser |
| [Brat](http://brat.nlplab.org/) | 2012 | 2016 | MIT License | Y | TXT,ANN | TXT,ANN,SVG,PNG | N | Y | Y | Y | Y | Y (DA) | N (document comparison) | SA, Web | Python, Apache |
| [Callisto](http://mitre.github.io/callisto/) | 2003 | 2013 | MITRE Propietary License | Y | APF | XML, SGML,AIF, APF | N | Y | N | Y | Y | Y (TA) | N | SA | Java 6 |
| [Egas](https://demo.bmd-software.com/egas/) | 2014 | 2016 | Download not available | Y | TXT, A1, BIOC | A1,BIOC | N | Y (max. 14 colours) | Y | Y | Y | Y (DA) | Y (EA) | N | Web browser |
| [GATE Teamware](https://gate.ac.uk/teamware/) | 2013 | 2015 | AGPL | Y | HTM, XML, CSV,TXT,RTF,PDF | XML | N | Y | Y | Y | Y | Y (TA) | Y(EA) | Web,SA | Java, MySQL |
| [Knowtator](http://knowtator.sourceforge.net/) | 2006 | 2010 | Mozilla Public License 1.1 | Y | XML | XML | N | Y | Y | N | N | Y (TA) | Y (EA) | SA | Java, Protege |
| [Markyt](http://www.markyt.org/) | 2014 | **2017** | **GNU GPL3** | **Y** | **TXT, HTML, BIOC,ANN,A1** | **HTML,BIOC, TSV,JSON** | **Y** | **Y** | **Y** | **Y** | **Y** | **Y (DA,TA)** | **Y (** EA, RA**)** | **SA, Web** | **MySQL, PHP, Java, Web browser** |
| [MAT](http://mat-annotation.sourceforge.net/) | 2012 | 2017 | BSD license | Y | JSON | CSV, XML, JSON | N | Y | Y | Y | Y | Y (TA) | Y(EA) | Web, SA | Python, Java, GNU |
| [Pubtator](https://www.ncbi.nlm.nih.gov/CBBresearch/Lu/Demo/PubTator/) | 2012 | 2013 | Download not available | N | TXT | XML | N | Y(max. 144 colours) | N | N | N | Y(TA) | N | N | Web browser |

**Legend:** Y- yes; N – no; NPM – node package manager; SA – stand alone, TA – table annotation, DA – document annotation, EA –Entity agreement, RA – Relation agreement. **^1^**Allow annotate raw HTML

# Tool references

In the table, time reference is two-fold: the year of initial publication; and, the year of the last update, if the tool is available to download, or the year of the last publication.

# A.nnotate

- Official webpage:
  - <http://a.nnotate.com/about.html>
- First publication:
  - Anagnostopoulou, C., Anagnostopoulou, C., & Howell, F. (n.d.). Collaborative online annotation of musical scores for eLearning using A.nnotate.com. Retrieved [02-11-2017] from http://citeseerx.ist.psu.edu/viewdoc/summary?doi=10.1.1.521.7992
  - International Technology, E. and D. C. (3 : 2009 : V., Gómez Chova, L., Martí Belenguer, D., & Candel Torres, I. (2009). *INTED 2009 proceedings cd*. *INTED2009 Proceedings*. International Association of Technology, Education and Development. Retrieved from https://library.iated.org/view/ANAGNOSTOPOULOU2009COL
- Download page:
  - <https://www.annotate.co/pricing.php>

# Anotation-Studio

- Official webpage:
  - <http://www.annotationstudio.org/news/updates/>
- First publication:
  - Paradis, J., Fendt, K., Kelley, W., Folsom, J., Gursoy, A., & Graham, E. (n.d.). Annotation Studio: Bringing a time-­‐honored learning practice into the digital age. Retrieved from http://hyperstudio.mit.edu/wp-content/uploads/2016/03/AS-NEHWhitePaper-6-25-complete1.pdf
- **Repository/Download page**:
  - <https://github.com/hyperstudio/Annotation-Studio>

# Argo

- Official webpage:
  - <http://argo.nactem.ac.uk/about-argo/>
- First publication:
  - Rak, R., Rowley, A., Black, W., & Ananiadou, S. (2012). Argo: an integrative, interactive, text mining-based workbench supporting curation. *Database : The Journal of Biological Databases and Curation*, *2012*, bas010. https://doi.org/10.1093/database/bas010
- Latest publication:
  - Rak, R., Carter, J., Rowley, A., Batista-Navarro, T., & Ananiadou, S. (n.d.). Interoperability and Customisation of Annotation Schemata in Argo. Retrieved from http://www.lrec-conf.org/proceedings/lrec2014/pdf/1086_Paper.pdf
- Download page: Not standalone tool, i.e. only usable from the official webpage.

# Brat

- Official webpage:
  - <http://brat.nlplab.org/>
- First publication:
  - Stenetorp, P., Pyysalo, S., Topić, G., Ohta, T., Ananiadou, S., & Tsujii, J. (2012). BRAT: a web-based tool for NLP-assisted text annotation. *Proceedings of the Demonstrations at the 13th Conference of the European Chapter of the Association for Computational Linguistics*. Association for Computational Linguistics. Retrieved from https://dl.acm.org/citation.cfm?id=2380942
- Download page:
  - <https://github.com/nlplab/brat>

# Callisto

- Official webpage:
  - <http://mitre.github.io/callisto/>
- First publication:
  - Day, D., Mchenry, C., Kozierok, R., & Riek, L. (n.d.). Callisto: A Configurable Annotation Workbench. Retrieved from http://www.lrec-conf.org/proceedings/lrec2004/pdf/612.pdf
- Download page:
  - <http://mitre.github.io/callisto/download.html>

# Egas

- Official webpage:
  - <https://demo.bmd-software.com/egas/>
- First publication:
  - Campos, D., Lourenco, J., Matos, S., & Oliveira, J. L. (2014). Egas: a collaborative and interactive document curation platform. *Database*, *2014*(0), bau048-bau048. https://doi.org/10.1093/database/bau048
- Latest publication:
  - Matos, S., Campos, D., Pinho, R., Silva, R. M., Mort, M., Cooper, D. N., & Oliveira, J. L. (2016). Mining clinical attributes of genomic variants through assisted literature curation in Egas. *Database*, *2016*, baw096. https://doi.org/10.1093/database/baw096
- Download page: Not standalone tool, i.e. only usable from the official webpage.

# GATE Teamware

- Official webpage:
  - <https://gate.ac.uk/teamware/>
- First publication:
  - Bontcheva, K., Cunningham, H., Roberts, I., Roberts, A., Tablan, V., Aswani, N., & Gorrell, G. (2013). GATE Teamware: a web-based, collaborative text annotation framework. *Language Resources and Evaluation*, *47*(4), 1007–1029. https://doi.org/10.1007/s10579-013-9215-6
- Download page:
  - <https://sourceforge.net/p/gate/code/HEAD/tree/teamware/>

# Knowtator

- Official webpage:
  - [http://knowtator.sourceforge.net](http://knowtator.sourceforge.net/)
- First publication:
  - Ogren, P. V., & V., P. (2006). Knowtator. In *Proceedings of the 2006 Conference of the North American Chapter of the Association for Computational Linguistics on Human Language Technology companion volume: demonstrations -* (pp. 273–275). Morristown, NJ, USA: Association for Computational Linguistics. https://doi.org/10.3115/1225785.1225791
- Download page:
  - <https://sourceforge.net/projects/knowtator/files/>

# Markyt

- Official webpage:
  - [http://www.markyt.org](http://www.markyt.org/)
- First publication:
  - Marky: A tool supporting annotation consistency in multi-user and iterative document annotation projects. (2015). *Computer Methods and Programs in Biomedicine*, *118*(2), 242–251. https://doi.org/10.1016/J.CMPB.2014.11.005
- Download page:
  - <https://github.com/sing-group/Markyt>

# MAT (The MITRE Annotation Toolkit)

- Official webpage:
  - <http://mat-annotation.sourceforge.net/>
- First publication/reference:
  - Clancy, S., Bayer, S., & Kozierok, R. (2012). Active Learning with a Human In The Loop. Retrieved [02-11-2017] from https://www.mitre.org/sites/default/files/pdf/12_4811.pdf - Page 7
- Download page:
  - <https://sourceforge.net/projects/mat-annotation/>

# Pubtator

- Official webpage:
  - <https://www.ncbi.nlm.nih.gov/CBBresearch/Lu/Demo/PubTator/>
- First publication:
  - Wei, C.-H., Kao, H.-Y., & Lu, Z. (2012). PubTator: A PubMed-like interactive curation system for document triage and literature curation. Retrieved from https://www.ncbi.nlm.nih.gov/CBBresearch/Lu/Demo/PubTator/tutorial/PubTator.pdf
- Last update:
  - Wei, C.-H., Kao, H.-Y., & Lu, Z. (2013). PubTator: a web-based text mining tool for assisting biocuration. *Nucleic Acids Research*, *41*(W1), W518–W522. https://doi.org/10.1093/nar/gkt441
- Repository/Download page: Not standalone tool, i.e. only usable from the official webpage.
