# Supplementary material for: Collaborative relation annotation and quality analysis in Markyt environment
Source: Database (Oxford). 2017 Dec 5;2017:bax090. doi: 10.1093/database/bax090 (PMC5737204; doi:10.1093/database/bax090)
Supplement: Supplementary Data 2 [file bax090_supplementary_material_2.docx]

*Supplementary material 2*: Annotation process in Markyt system

Collaborative relation annotation and quality analysis in Markyt environment

Martín Pérez-Pérez, Gael Pérez-Rodríguez, Florentino Fdez-Riverola, Anália Lourenço^§^

^§^Corresponding author

# Entity and relation annotation in Markyt

This supplementary material details how to perform the annotation of entities and relations in Markyt system. The user may choose between the mention and the document level annotation perspectives, which are interchangeable at the project level without preserving the annotations of relations, i.e. the annotated relations will be deleted.

Note that, before starting the annotation process, the administrator must create the types for entities and relations.

## Annotations using inline perspective

This is the most conventional annotation perspective and it is useful for most annotation projects. At the mention level both entities and relations are annotated in the text. The annotation procedure is mouse-based and uses intuitive gestures familiar from text editors, presentation software, and many other tools. First, the annotator has to select the entity or relation type from the left-side panel (Figure 1).


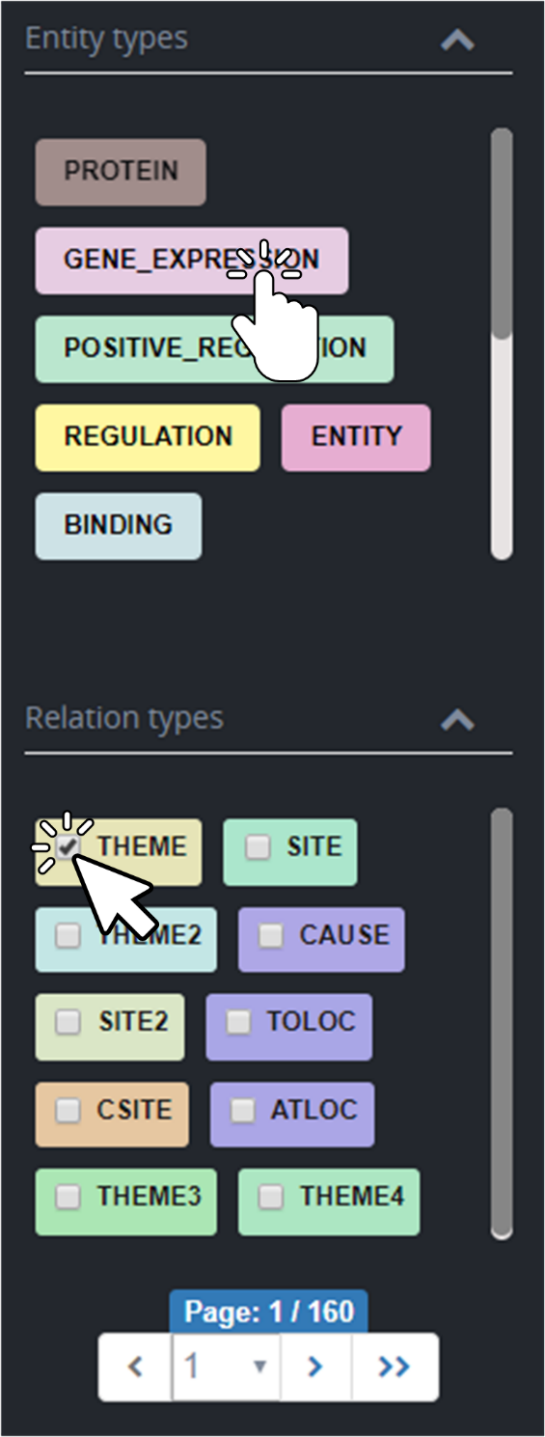


**Figure 1**. Selection of entity or relation types in the annotation interface.

Then, to mark a span of text, the annotator simply selects it with the mouse by dragging or by double-clicking on the text (Figure 2).


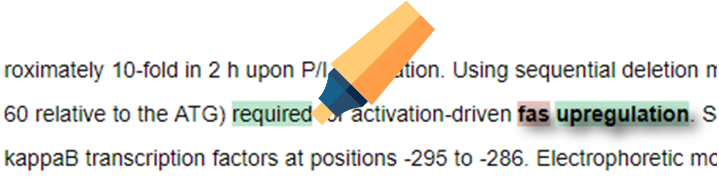


**Figure 2**. Selecting text for the annotation of an entity.

When describing a relation, the annotator selects the corresponding type and then, left-clicks on one entity and drags the relation (represented by a line or arrow) to the other (Figure 3).


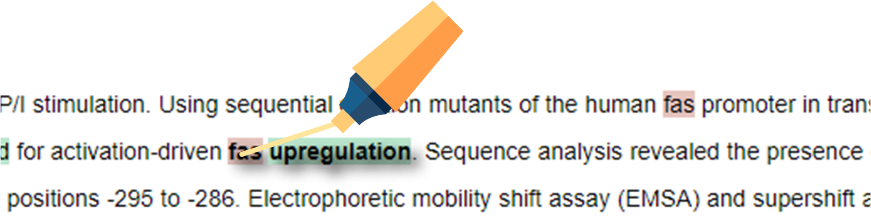


**Figure 3**. Connecting entities to create a relation.

## Annotations using stand-off perspective

This perspective of annotation was designed with the purpose of enabling the annotation of relations without requiring local textual contextualisation, i.e. specific text offsets. For example, it is useful to generate co-occurrence corpora, i.e. there can only be one relation per type between two identical entities. It is important to remark, that in this level is not possible to annotate entities, so, the documents must have the annotated terms before the generation of these relations.

At this level, annotation procedure is table-based and uses simple checkboxes and combo boxes to select the information. Before creating the relations, the annotator must select the types of the entities that will be related from the left-side panel (Figure 4).


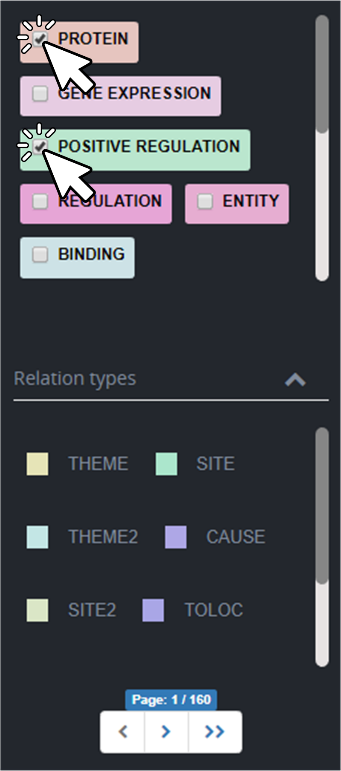


**Figure 4**. Entity types selection in the interface panel.

After selecting the desired entity types, a relationship table will appear below each document that has at least one entity of each selected type (Figure 5). It is important to remark that by default every row presented in this perspective show plausible but not accepted relations between two entities.


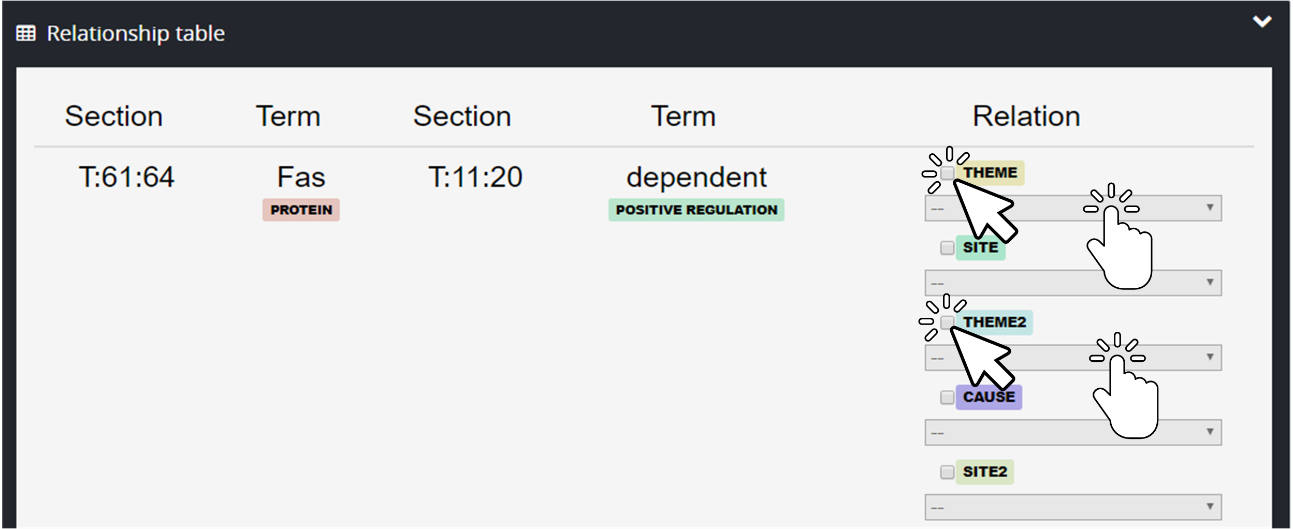


**Figure 5**. Creating relations using the relationship table.

As illustrated in the previous figure, the annotator can generate more than one relation per entity pair (e.g. “Fas” term will be related twice with “dependent” term with the relation types “theme” and “theme 2”). The combo box next to the relation type enables the annotator to specify which is the target of the relation (i.e. if relation is directed).
