# Supplementary material for: Collaborative relation annotation and quality analysis in Markyt environment
Source: Database (Oxford). 2017 Dec 5;2017:bax090. doi: 10.1093/database/bax090 (PMC5737204; doi:10.1093/database/bax090)
Supplement: Supplementary Data 3 [file bax090_supplementary_material_3.docx]

*Supplementary material 3*: Annotation interface in Markyt system

Collaborative relation annotation and quality analysis in Markyt environment

Martín Pérez-Pérez, Gael Pérez-Rodríguez, Florentino Fdez-Riverola, Anália Lourenço^§^

^§^Corresponding author

# Annotation interface and main capabilities in Markyt

This supplementary material presents the supporting functionalities that the annotator has available in the inline annotation perspective (Figure 1).


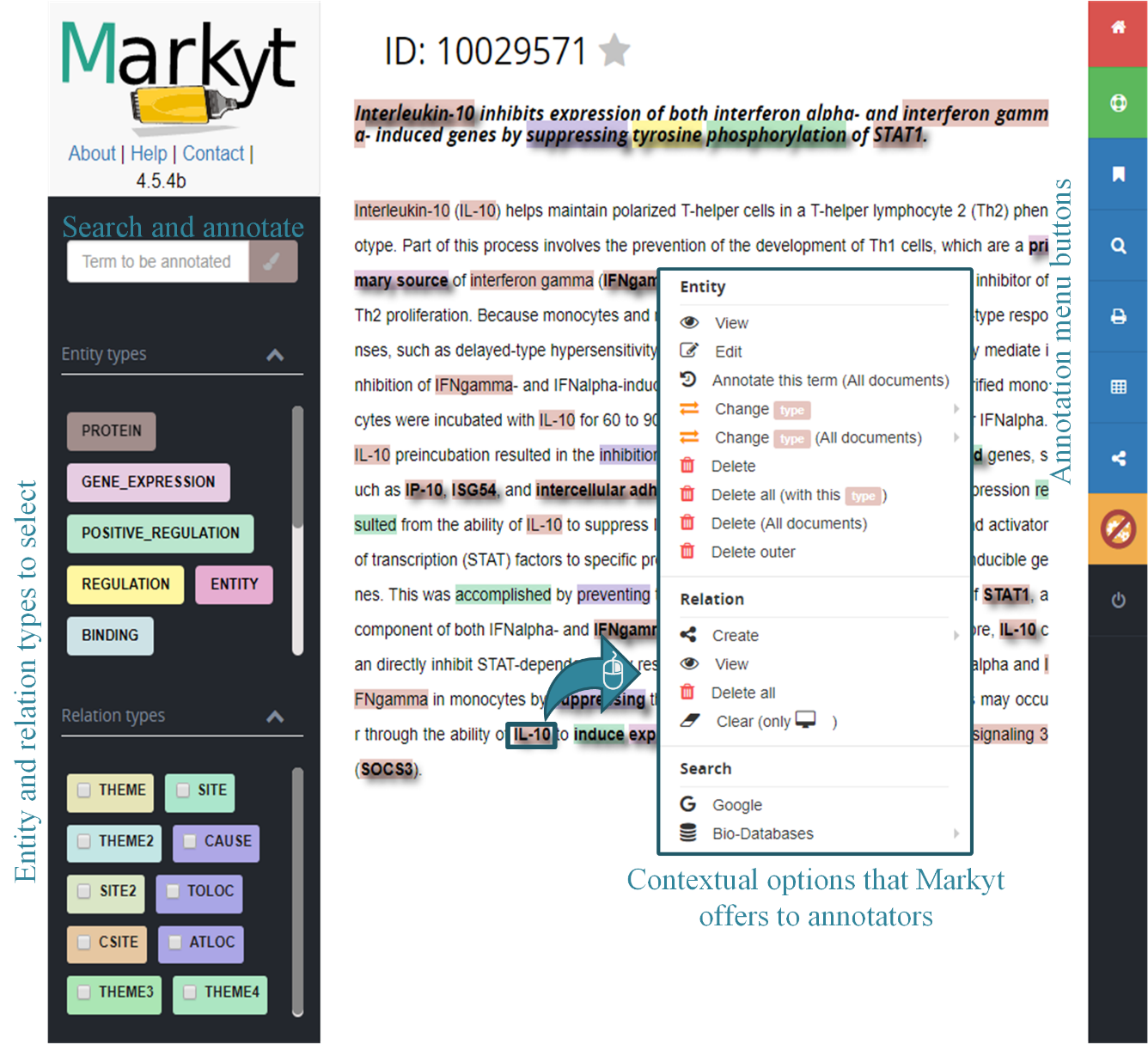


**Figure 1**. Illustration of the inline perspective with its main capabilities. The left-hand side menu details the entity and relation types. The right-hand side enables several search and visualization options. Entity-specific edition menu is prompted by right-click mouse action.

## Left-side menu operations

In this menu there are two main panels: a search option to help track down terms to be annotated, and a panel listing all the entity and relation types available for the current round of the project (Figure 2).


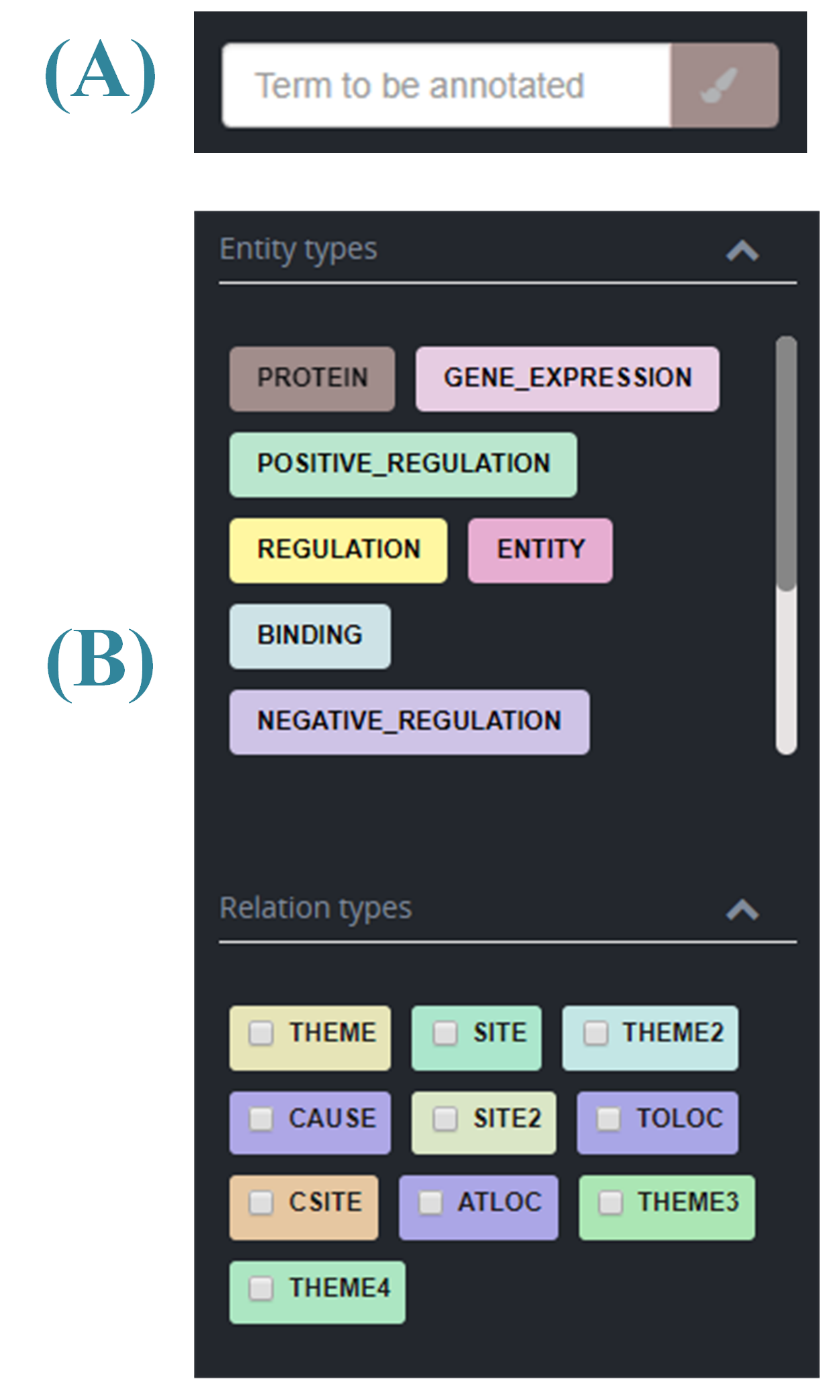


**Figure 2**. Illustration of the left-side menu panel operations. (A) is the field to search and annotate entities. (B) is the panel of available entity and relation types.

The search field enables the annotator to introduce a single term to be annotated in all documents of the corpus (Figure 2A). The annotator must select one type per entity and relation to start the annotation process (Figure 2B). For example, if the annotator selects the entity type “Regulation”, it will be possible to make annotations with that type in the text.

## Right-side bar operations

There are several buttons grouped by colours based on their functionalities in this bar. The explanation of these buttons following an up to down order is provided below:

- The bookmark button lets the annotator navigate to the desired document (Figure 3). The field has an autocomplete option so it is only necessary to introduce part of the document ID.


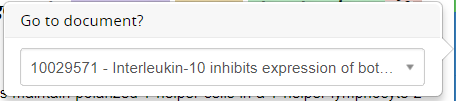


**Figure 3**. Document navigation field text.

- The magnifying glass button lets the annotator search any annotated entity in all documents (Figure 4). The results of the search will be shown in the lower right corner of the screen.


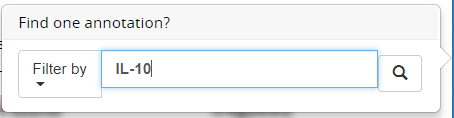


**Figure 4**. Annotated entity search field.

- The printer button lets the annotator print all the documents in the current page. The annotated entities and relations will appear in the printed documents.
- The table button lets the annotator display the inline table of relations for mention level projects. This table is useful to search, display and delete annotated relations of the current page (Figure 5).


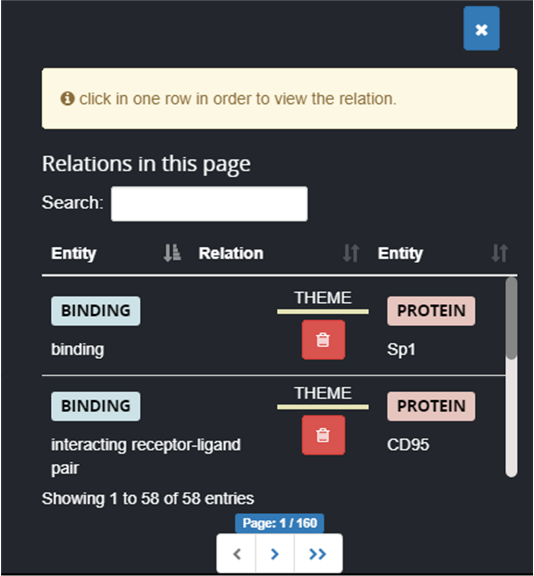


**Figure 5**. Reduced version of the inline relation table.

- The network button lets the annotator display the relations directly in the text of the documents. This view will draw the relations as solid lines between the two related entities.

## Contextual menu options

This contextual menu can be popped up by right-clicking in any annotated entity. In this menu, the operations are grouped into entity, relation and search categories (Figure 6).


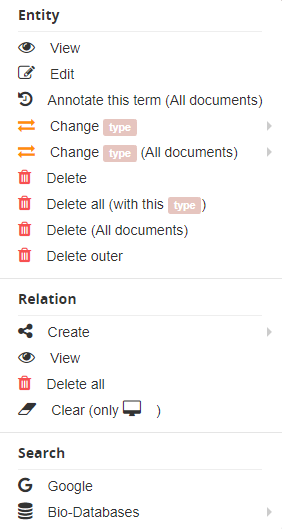


**Figure 6**. Operations in the contextual menu.

Most entity-centred options are self-explanatory like “Delete…” and “Change type”. The “View” and “Edit” operations deal with questions/comments associated to the entity, i.e. the questions/comments specified by the administrator when configuring the entity types (e.g. the request of a normalization identifier). The “Delete outer” refers to the capability of eliminating the parent annotations in overlapped annotations (see example in Figure 7).


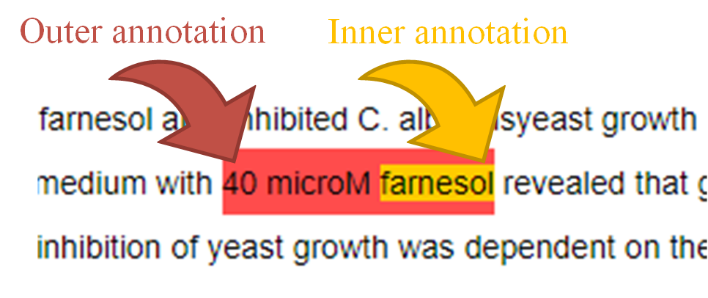


**Figure 7**. Example of an overlapped annotation.

In terms of relation-centred operations, it is possible to connect two annotated entities using "Create". The annotator has to select one of the available relation types that will appear after selecting this option and then the annotator must select another entity in the document using the left-click button of the mouse. The "Delete all" operation removes all the relations containing the selected entity in the document. "View" enables the visualisation of all relations at the current page, whereas “Clear” disables it. This relation will be represented using a solid line with the colour of the relation type.

The search capabilities are useful to search for the selected entity in the WWW. Markyt supports searches in Google and in several well-known biomedical databases (e.g. DrugBank, Uniprot and PubChem).
